# Non-Alcoholic Fatty Liver Disease Is Closely Associated with Sub-Clinical Inflammation: A Case-Control Study on Asian Indians in North India

**DOI:** 10.1371/journal.pone.0049286

**Published:** 2013-01-11

**Authors:** Priyanka Nigam, Surya P. Bhatt, Anoop Misra, Meera Vaidya, Jharna Dasgupta, Davinder Singh Chadha

**Affiliations:** 1 National Diabetes Obesity and Cholesterol Foundation (N-DOC), SDA, New Delhi, India; 2 Department of Food and Nutrition, Govt. M. H. College of Home Science and Science for Woman, Jabalpur, India; 3 Department of Medicine, All India Institute of Medical Sciences, New Delhi, India; 4 Fortis-CDOC Center of Excellence for Diabetes, Metabolic Diseases and Endocrinology, B 16, Chirag Enclave, New Delhi, India; 5 Department of Cardiology, MH (CTC), AFMC, Pune, India; 6 Diabetes Foundation (India), New Delhi, India; University of Leicester, United Kingdom

## Abstract

**Objectives:**

Association between sub-clinical inflammation and non-alcoholic fatty liver disease (NAFLD) has not been studied in Asian Indians. In this case-control study, we aimed to analyse association of NAFLD with the sub-clinical inflammation and metabolic profile in Asian Indians in north India.

**Methods:**

Ultrasound diagnosed 120 cases of NAFLD were compared to 152 healthy controls without NAFLD. Anthropometric profile [body mass index (BMI), waist circumference (WC), hip circumference (HC)], high-sensitivity C-reactive protein (hs-CRP), metabolic profile [fasting blood glucose (FBG), lipid profile] and hepatic function tests [alanine aminotransferase (ALT) and aspartate aminotransferase (AST)] were recorded.

**Results:**

Metabolic parameters [FBG, total cholesterol (TC), serum triglycerides (TG),low-density lipoprotein (LDL-c)], hs-CRP and prevalence of the metabolic syndrome were higher in cases as compared to controls (p-value<0.05 for all). The median (range) of hs-CRP (mg/L) for cases [2.6(0.2–13.4)] were significantly higher than in controls [1.4(0.03–11.4), p = 0.01]. Similarly, higher values of hs-CRP were obtained when subgroups of cases with obesity, abdominal obesity and the metabolic syndrome were compared to controls [2.75 (0.03–14.3) *vs.* 1.52 (0.04–14.3), p = 0.0010; 2.8 (0.03–14.3) *vs.* 1.5 (0.06–14.3), p = 0.0014 and 2.7 (0.5–14.3) *vs.* 1.6 (0.06–8.5), p = 0.0013, respectively. On multivariate logistic regression analysis BMI (p = 0.001), WC (p = 0.001), FBG (p = 0.002), TC (p = 0.008), TG (p = 0.002), blood pressure (p = 0.005), metabolic syndrome (p = 0.001) and hs-CRP (p = 0.003) were significantly and independently associated with NAFLD. After adjusting for significant variables, the association between high hs-CRP and NAFLD remained large and statistically significant [adjusted OR = 1.17, 95% confidence interval (CI) = 1.05–1.29]. An increase in 1 mg/dl of hs-CRP level calculated to increase the risk of developing NAFLD by 1.7 times as compared to controls after adjusting for significant variables associated with NAFLD.

**Conclusions:**

In this cohort of Asian Indians in North India, presence of NAFLD showed independent relationships with sub-clinical inflammation.

## Introduction

High sensitivity C-reactive protein (hs-CRP), synthesized in hepatocytes, is an acute-phase reactant that increases non-specifically in bacterial infections, immuno-inflammatory diseases and malignant disorders. Obesity, particularly abdominal adiposity, is characterised by low-grade systemic inflammation. In prospective studies, high hs-CRP levels have been shown to predict the metabolic syndrome [1], type 2 diabetes mellitus (T2DM) [2] and coronary heart disease (CHD) [3]. Increased hs-CRP levels have been shown to correlate with generalised and abdominal adiposity in Asian Indians [4].

The development of non-alcoholic fatty liver disease (NAFLD) is strongly associated with the presence of the metabolic syndrome [5]. Current understanding of pathogenesis of NAFLD and non-alcoholic steato-hepatitis (NASH) involves a two-hit hypothesis wherein first, hepatic insulin resistance causes steatosis, and second, pathogenic stimulus causes oxidative stress and cytokines production leads to hepatic inflammation. Interestingly, systemic sub-clinical inflammation could be contributed by hepatic inflammation as well as from visceral adipose tissue. Recent data also show that hs-CRP is a biomarker for NAFLD in some ethnic groups (Japanese) [6] while no association has been shown by others (Europeans) [7].

Asian Indians are highly predisposed to develop insulin resistance, the metabolic syndrome, T2DM and CHD; more than white Caucasians [8], [9]. Asian Indians have abnormal body composition consisting of high body fat and abdominal adiposity that may partially explain the high prevalence of these clinical disorders [8]. Further, they have higher hs-CRP levels than white Caucasians [10], [11]. Our previous studies have shown high hs-CRP levels in urban Asian Indian adolescents living in India [4]. Further, we also showed that accumulation of fat in other ectopic sites (soleus muscles; intra-myocellular triglycerides) showed correlation with hs-CRP but not insulin resistance [12].

There is paucity of data regarding sub-clinical inflammation and NAFLD in Asian Indians. We hypothesized that sub-clinical inflammation is closely correlated with NAFLD among Asian Indians. To test this hypothesis we designed a case (subjects with NAFLD) and controls (subjects without NAFLD) by analyzing anthropometric and metabolic profiles and hs-CRP levels.

## Subjects and Methods

### Ethics Statement

This study has been done in Northern part of India at Fortis Hospital, New Delhi from February 2006 to June 2008 after approval from the institutional ethics committee. All subjects gave written informed consent. Subjects were recruited from the outpatient department of Fortis Hospital.

Subjects with significant alcohol intake (>20 g/day) type 2 diabetes mellitus (T2DM), cardiovascular disease (CVD), presence of other liver diseases (alcoholic liver disease, viral hepatitis, autoimmune hepatitis, primary biliary cirrhosis, biliary obstruction, drug-induced liver damage etc.), severe end organ damage, human immunodeficiency virus infection, pregnancy and lactation, were excluded from the study. A detailed history (demographic and social economic profiles, history of smoking, and alcohol intake and physical activity patterns) and family history (T2DM, overweight, hypertension, liver disease, and CVD) were obtained. Clinical, demographic and socioeconomic profiles of 272 apparently healthy subjects were studied.

For measurement of weight, subject was instructed to stand still in the platform, with the body weight evenly distributed between both the feet. After removing heavy clothing weight was measured to the nearest of 0.1 kg. Height was measured using stadiometer with head held in Frankfort plane to the nearest of 0.1 cm. Body mass index (BMI) was calculated by the following formula; weight (kg)/height (m^2^). Waist circumference (WC) was measured mid way between iliac crest and lowermost margin of the ribs, in quiet breathing. Hip circumference (HC) was measured at the maximum protruding part of buttocks at the level of the greater trochanter with the patient wearing minimal clothing and with feet together. Mid-thigh circumference was taken at the point in anterior midline of the thigh, midway between the inguinal ligament and base of patella to the nearest of 0.1 mm. Pulse rate was recorded after 5 minutes of rest. Blood pressure was also recorded after at least 5 minutes of rest in a chair, with feet on the floor, and arm supported at heart level, using a mercury sphygmomanometer. An appropriate-sized cuff (cuff bladder encircling at least 80% of the arm) was used to ensure accuracy. Systolic blood pressure was measured at the point where the first of two or more sounds was heard (phase 1), and diastolic blood pressure before the disappearance of sounds (phase 5).

### Biochemical Analysis and hs-CRP Assay

Estimations for fasting blood glucose (FBG), total cholesterol (TC), serum triglycerides (TG), high-density lipoprotein cholesterol (HDL-c), low-density lipoprotein cholesterol (LDL-c), aspartate aminotransferase (AST) and alanine aminotransferase (ALT) levels were done as previously described [13].

Levels of hs-CRP were measured using ELISA kit (Biocheck Inc., CA, USA). In this assay system unique monoclonal antibody was directed against a distinct antigenic determinant on the CRP molecule and it is attached to the surface of microtitre wells on the ELISA plate. A horseradish peroxidase-conjugated goat anti-CRP antibody was used as the secondary antibody was used as the secondary antibody. The test sample was allowed to react simultaneously with the antibodies resulting in sandwiching of CRP molecules between solid phase and enzyme linked antibodies. Intensity of the blue color following addition of tetramethylbenzidine substrate is directly proportional to the concentrations of CRP in the sample. According to the manufacturers, the normal CRP concentration using this assay ranged from 0.068 to 8.2 mg/l, the lowest detectable limit being 0.005 mg/l. The intra-assay variation determined using duplicate samples was 1.7–3.3%.

### Ultrasound Imaging of Liver

Liver ultrasound was carried out using 3.5 MHz curvilinear probe (Siemens-G 60 S 2004, Germany) by a trained radiologist with post graduate qualifications, who followed the standardized procedure. A complete examination required both sub-costal and inter-costal scanning. The definition of fatty liver was based on a comparative assessment of image brightness relative to the kidneys, according to previously reported diagnostic criteria [14]–[16]. Severity of fatty liver was classified according to the brightness compared to kidneys, blurring of gall bladder wall and attenuation of hepatic veins. Liver span was measured in the mid-clavicular line by marking upper and lower limit of liver using ultrasonic probe. The radiologist performing the ultrasound was blinded to the clinical data.

### Definitions

High hs-CRP level was defined as >1 mg/L [17]. Overweight/obesity was defined as BMI ≥23 kg/m^2^ [18]. Waist circumference ≥90 cm for males and ≥80 cm for females was considered an indicator of abdominal obesity [19]. Impaired fasting glucose (IFG) was diagnosed according to the diagnostic criteria of the American Diabetes Association [20]. The metabolic syndrome was defined based on the consensus statement for diagnosis of obesity, abdominal obesity and metabolic syndrome for Asian Indians [18]. Physical activity is defined as >20 minutes brisk walk per day. Any degree of current smoking was taken as definition of smoking.

### Statistical Analysis

Data were presented as either mean ± standard deviation (SD) or median (range) as appropriate. The differences in mean values of the variables between cases and controls were tested using student t-test. Chi square test was used to test the association between categorized variables and independent t-test was used to compare means of continuous variables. Wilcoxon rank-sum test was used to detect the differences in hs-CRP values between cases and controls with obesity, abdominal obesity, and metabolic syndrome. Multivariate logistic regression analysis was carried out with NAFLD as the dependent variable and smoking, family history of T2DM and CVD, age, BMI, WC, TC, FBG, HDL-c, LDL-c, ALT, AST and hs-CRP (after removing the outliers to achieve normality) as an independent variables to assess significant predictors and unadjusted odds ratio and 95% confidence interval (OR (95% CI). To assess the effect of hs-CRP on NAFLD, adjusted odds ratio estimated by controlling the other significant predictors of NAFLD. p-value<0.05 was considered as statistically significant. Various statistical measures evaluated with the help of statistical packages SPSS 11 for windows (SPSS, Inc., Chicago, IL, USA).

## Results

Among 272, 120 subjects (males 92) had NAFLD (“cases”) while 152 subjects (males 105) had normal liver ultrasonography (“controls”). Smoking and family history of T2DM and CVD were significantly higher among cases as compared to controls (Table 1). Prevalence of overweight, obesity, the metabolic syndrome, hs-CRP levels and IFG were significantly higher in cases than controls (Table 2). Significantly higher values of BMI, WC, HC, weight- to-height ratio, mid-thigh circumference, FBG, TC, TG, LDL-c, AST and ALT were recorded in cases than in controls (Table 3).

**Table 1:** Demographic and Lifestyle Profiles.

*Variables*	*Cases (n = 120)*	*Controls (n = 152)*	*p value*
Sex (Male) n (%)	92 (76.7)	105 (69.1)	
Education n (%)			
Illiterate	2 (5.1)	5 (6.0)	
Primary (class 1^st^ to 9^th^)	3 (7.6)	6 (7.3)	
Intermediate (class 10^th^–12^th^)	12 (30.7)	32 (39.0)	0.94
Graduate	20 (51.3)	35 (42.7)	
Post graduate	2 (5.1)	4 (4.9)	
Family history n (%)			
Type 2 diabetes mellitus	48 (28.48)	43 (40)	0.05
Hypertension	34 (23.2)	35 (28.3)	0.33
Overweight	30 (25)	28 (18.5)	0.19
Cardiovascular disease	14 (11.7)	6 (4)	0.01
Physical activity n (%)			
Never	59 (49.2)	82 (54.7)	
Once a week	4 (3.3)	6 (4.0)	0.39
Two-three times in a week	11 (7.3)	9 (7.5)	
Daily	48 (40)	48 (32)	
Smoking	22 (18.3)	15 (10.0)	0.04

**Table 2:** Distribution of hs-CRP According to Presence of Impaired Fasting Glucose, Body Mass Index and the Metabolic Syndrome.

*Variables n (%)*	*Cases (n = 120)*	*Controls (n = 152)*
BMI (kg/m^2^)*		
<18.9	2 (1.7)	17 (11.4)
19–22.9	6 (5.1)	28 (18.6)
23–24.9	15 (12.7)	27 (18)
>25	95 (80.5)	78 (52)
The metabolic syndrome*	76 (66.09)	39 (33.91)
hs-C-reactive protein (mg/L)*		
<1	28 (23.2)	64 (41.8)
1–2.9	39 (32.2)	47 (30.7)
>3	54 (44.6)	42(27.5)
Impaired fasting glucose*	16 (13.3)	6 (3.97)

* p<0.05.

**Table 3:** Clinical and Biochemical Parameters.

*Variable*	*Cases (Mean ± SD) (n = 120)*	*Controls (Mean ± SD) (n = 152)*	*p-value*
Age (y)	38.5±8.8	36.2±10.8	0.06
Pulse rate (per min)	79.2±7.4	78.2±7.7	0.27
Systolic blood pressure (mmHg)	126.20±15.0	123.02±13.15	0.06
Diastolic blood pressure (mmHg)	80.57±9.7	79.25±9.04	0.25
Body mass index (kg/m^2^)	27.9±3.6	24.7±4.2	0.01
Waist circumference (cm)	93.55±11.12	86.0±16.04	0.01
Hip circumference (cm)	98.1±8.6	92.6±11.5	0.01
Weight-to-height ratio	34.5±4.80	32.0±6.36	0.01
Mid-thigh circumference (cm)	54.6±9.09	49.93±8.04	0.01
Fasting blood glucose (mg/dl)	91.31±19.4	84.83±9.31	0.001
hs-C-reactive protein (mg/L)*	2.6 (0.23, 13.4)	1.42 (0.03,11.4)	0.01
Total cholesterol (mg/dl)	182.74±31.94	164.24±32.63	0.01
Serum triglycerides (mg/dl)	166.36±78.85	134.95±78.35	0.001
HDL-c (mg/dl)	41.31±7.2	41.98±6.54	0.43
LDL-c (mg/dl)	106.8±24.1	96.8±25.3	0.001
Aspartate aminotransferase (U/l)	41.58±31.52	32.90±18.57	0.005
Alanine aminotransferase (U/l)	36.15±22.69	30.16±11.42	0.005

* median (range).

**Figure 1:**
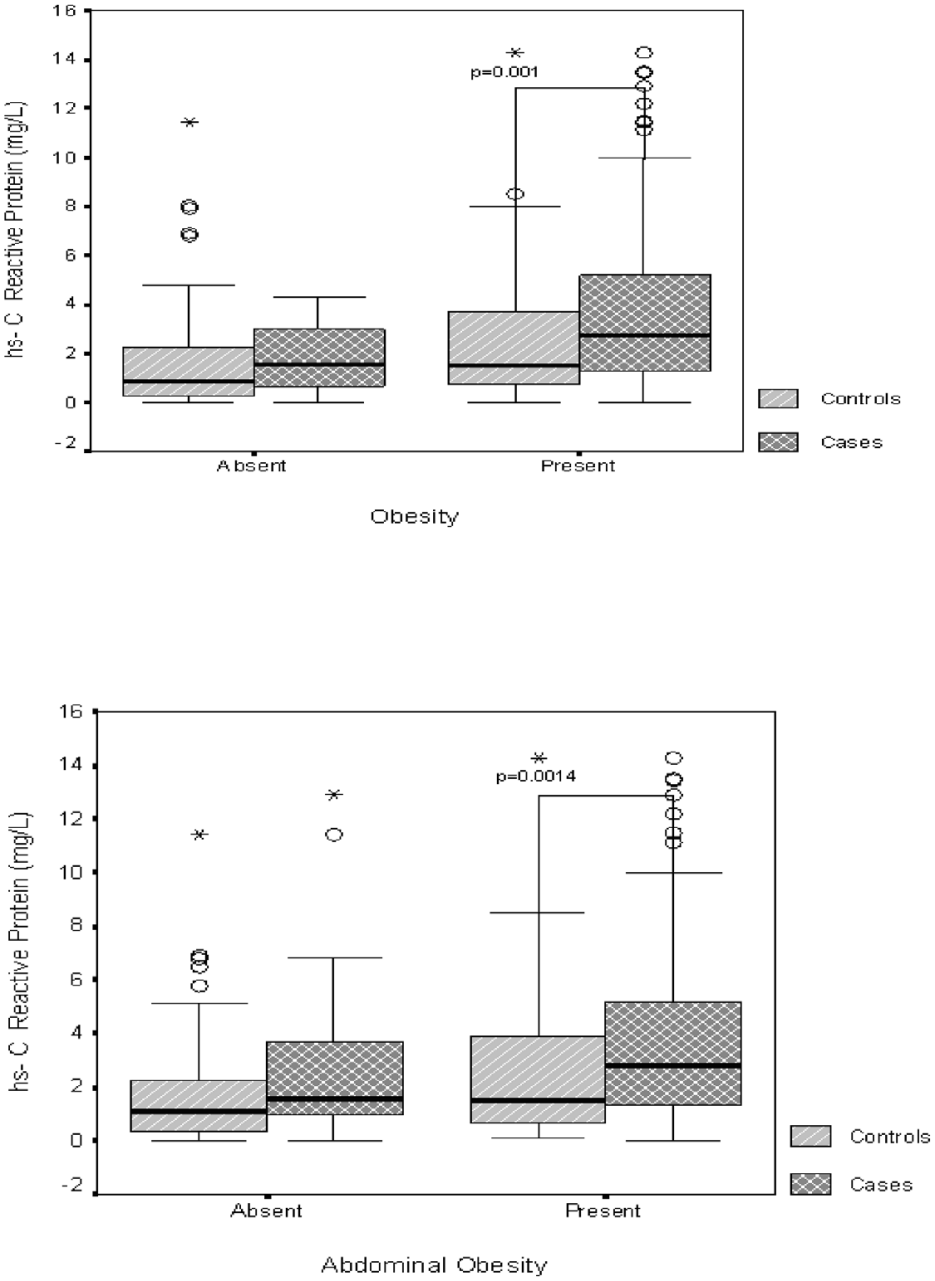
Box plot representation of hs-CRP levels in subjects with non-alcoholic fatty liver disease and in controls having overweight and obesity (a), abdominal obesity (b), and the metabolic syndrome (c). Each box comprises the values between the 25^th^ and the 75^th^ percentiles, and the bold horizontal line is the median value; the whiskers stretch from the 10^th^ and to the 90^th^ percentile. Circles represent individual outlier's value. Stars represent extremes value of individual.

The median (range) of hs-CRP (mg/dL) for cases [2.6 (0.2–13.4)] was significantly higher than controls [1.4 (0.03–11.4), p value = 0.01] (Table 3). Similarly, higher values of hs-CRP were obtained when subgroups of cases and controls with obesity, abdominal obesity and the metabolic syndrome were compared [2.75 (0.03–14.3) *vs.* 1.52 (0.04–14.3), p = 0.001; 2.8 (0.03–14.3) *vs.* 1.5 (0.06–14.3), p = 0.0014; and 2.7 (0.5–14.3) *vs.* 1.6 (0.06–8.5), p = 0.0013, respectively]. (Fig. 1)

On multivariate logistic regression analysis BMI (p = 0.001), WC (p = 0.001), FBG (p = 0.002), TC (p = 0.008), TG (p = 0.002), blood pressure (p = 0.005), the metabolic syndrome (p = 0.001) and hs-CRP (p = 0.003) were significantly and independently associated with NAFLD (Table 4). To assess the effect of hs-CRP on NAFLD, adjusted OR (95% CI) for hs-CRP was estimated after adjusting for significant variables. Unadjusted and adjusted OR (95% CI) for hs-CRP were 1.2 (1.09–1.38) and 1.7 (1.05–1.29), respectively. An increase in 1 mg/dl of hs-CRP level calculated to increase the risk of developing NAFLD by 1.7 times as compared to controls after adjusting for significant variables associated with NAFLD.

**Table 4:** Logistic Regression Analysis with hs-CRP as the Outcome Variable and Anthropometric and Metabolic Variables as Co-variates.

*Variables*	*Cases n (%) n-120*	*Controls n (%) n = 152*	*p-value*	*Univariate OR (95% CI)*
BMI (kg/m^2^)				
≥23	112 (93.3)	106(70.2)	0.001	5.9
<23	8 (6.7)	45 (29.8)		(2.67, 3.19)
Waist circumference (cm)				
>80 for female/90 for male	102(85)	94 (62.3)	0.001	3.43
≤80 for female/90 for male	18 (15)	57 (37.7)		(1.88, 6.25)
Triglycerides (mg/dl)				
≥150	57 (48.3)	45 (30.2)	0.002	2.15
<150	63 (51.7)	104 (69.8)		(1.30, 3.56)
Total cholesterol (mg/dl)				
>200	31 (24.58)	18 (12.08)	0.008	2.37
≤200	89 (75.42)	131(87.92)		(1.24, 4.52)
Fasting blood glucose (mg/dl)				
>100	23 (18.6)	9 (6.1)	0.002	3.53
≤100	97 (83.4)	139 (93.9)		(1.56, 8.01)
HDL-c (mg/dl)				
≥40 for male/50 for female	34 (27.3)	49 (32.9)	0.330	0.7
<40 for male/50 for female	86 (72.6)	100 (67.1)		(0.45, 1.3)
Blood pressure (mmHg)				
≥130/85	93 (77.5)	93 (61.6)	0.005	1.10
<130/85	27 (22.5)	58 (38.4)		(0.77, 1.56)
hs-CRP (mg/L)				
≥1	94 (79)	94 (62.3)	0.003	1.2
<1	25 (21)	57 (37.7)		(1.1,1.3)
The metabolic syndrome				
Present	67 (57.3)	53 (35.8)	0.001	2.4
Absent	53 (42.7)	95 (64.2)		(1.4, 3.9)

## Discussion

This is the first case-control study in which comprehensive analysis of hs-CRP, anthropometric and metabolic co-variates has been researched among Asian Indians in north India. In this study, the NAFLD was associated with hs-CRP in apparently healthy Asian Indians independent of obesity and abdominal obesity.

Several studies have suggested the association of NAFLD with obesity, abdominal obesity, dysglycemia and various components of the metabolic syndrome [21]. However, limited data show elevation of serum hs-CRP levels. The current study has also shown higher hs-CRP levels in NAFLD as compared to those without NAFLD. Our observations are important in the light of the information that hs-CRP levels are higher in Asian Indians than in White Caucasians [11], [22]. The findings of present study are in line with previous data on cross-sectional association between the hs-CRP and NAFLD in Japanese and Korean Asians [23], [24]. In the cross-sectional study by Park *et al* [25], elevated hs-CRP level was associated with NAFLD in apparently healthy non-obese Korean men.

Elevation of serum hs-CRP usually reflects its synthesis in response to a pathological process [26]. *In vivo* release of interleukin-6 (IL-6), linked closely to hs-CRP pathway, but not tumor necrosis factor-α (TNF-α), which is related to insulin resistance, has been reported in human subcutaneous adipose tissue (SAT) [27]. We speculate that relatively larger SAT mass (truncal and peripheral SAT) in Asian Indians as compared to white Caucasians as has been shown in several studies [4], [8], is likely to generate relatively higher amounts of hs-CRP and preferentially drive this pathway rather than the insulin resistance pathway, although both appear to be interlinked. This is in line with our previous study [28] wherein triceps skin fold thickness (indicative of peripheral SAT) was an independent risk factor associated with elevated hs-CRP level. Importantly, when compared with white Caucasians and blacks, triceps skin fold thickness was significantly thicker in Asian Indians [29]. Further, hepatic triglycerides appear to be higher in Asian Indians as compared to white Caucasians. By using proton magnetic resonance spectroscopy, Shulman *et al* [30] measured hepatic triglyceride (HTG) content and plasma IL-6 concentrations in different ethnic groups in USA. Interestingly, these authors reported that the HTG content and plasma IL-6 concentrations were nearly 2-fold higher in Asian Indians as compared to white Caucasians. Whether this increased hepatic HTG content could lead to increased hs-CRP levels in Asian Indians has not been investigated.

While the pathophysiology of NAFLD remains incompletely understood, accumulation of triglycerides in hepatocytes in presence of oxidative stress, lipid peroxidation, pro-inflammatory cytokines (e.g. TNF-α, IL-6) appears to be important [31]. In the patients with NAFLD and NASH, liver biopsies have revealed hepatic distribution (mRNA) of the inflammatory cytokine TNF-α with its receptors [32] and the adiponectin with its receptors [33]. As showed in animal studies, the increased amount of fatty acids present in the liver may mediate hepatic production of TNF-α, causing increased systemic levels of TNF-α [34]. When hepatocytes get damaged, liver-specific macrophages (‘Kupffer Cells’) get activated and secrete more TNF-α and IL-6 into the blood [35]. TNF-α and IL-6 are considered to induce hepatic production of the acute phase protein hs-CRP [36].

A limitation of this study was that the diagnosis of NAFLD was based on liver ultrasonography. It has been argued that other methods; magnetic resonance spectroscopy and liver biopsy are better tools for defining NAFLD, and could be considered as “gold standards”. Conversely, ultrasonography is by far the most common method of diagnosing NAFLD in clinical practice and has a fair sensitivity (87%) and specificity (94%) in detecting hepatic steatosis [37]. It is simple to perform, non-invasive, cost-effective and does not entail any radiation hazard, and could also be used in the epidemiological studies. Previous publications in reference to hs-CRP in NAFLD have also relied either exclusively or substantially on ultrasound-based imaging for diagnosis of hepatic steatosis [38]. Hence, although not “gold standard”, this method of investigation provides reasonable alternative to more expensive and difficult-to-perform diagnostic methods of NAFLD.

In summary, we demonstrate the association of inflammatory response (hs-CRP) with NAFLD in Asian Indians in north India for the first time. High hs-CRP levels, dysglycemia, obesity, and abdominal obesity were found to be independent predictors of NAFLD.
